# PDE2 Inhibits PKA-Mediated Phosphorylation of TFAM to Promote Mitochondrial Ca^2+^-Induced Colorectal Cancer Growth

**DOI:** 10.3389/fonc.2021.663778

**Published:** 2021-06-21

**Authors:** Yilin Zhao, Yaya Wang, Jing Zhao, Zhaohui Zhang, Mingpeng Jin, Feng Zhou, Chao Jin, Jing Zhang, Jinliang Xing, Nan Wang, Xianli He, Tingting Ren

**Affiliations:** ^1^ Department of Clinical Oncology, Xijing Hospital, Fourth Military Medical University, Xi’an, China; ^2^ State Key Laboratory of Cancer Biology and Department of Physiology and Pathophysiology, Fourth Military Medical University, Xi’an, China; ^3^ College of Chemistry and Chemical Engineering, Xi’an University of Science and Technology, Xi’an, China; ^4^ Department of General Surgery, Tangdu Hospital, Fourth Military Medical University, Xi’an, China; ^5^ Department of General Surgery, Huaihai Hospital, Xuzhou Medical University, Xuzhou, China; ^6^ State Key Laboratory of Cancer Biology and Experimental Teaching Center of Basic Medicine, Fourth Military Medical University, Xi’an, China

**Keywords:** mitochondrial Ca^2+^, colorectal cancer, mitochondrial transcription factor A, mitochondrial calcium uniporter, phosphodiesterase 2

## Abstract

Growing evidence indicates that the dysregulation of mitochondrial calcium (Ca^2+^) plays a critical role in the growth of tumor cells, including colorectal cancer (CRC). However, the underling mechanism is not fully elucidated. In this study, the regulatory effects of mitochondrial Ca^2+^ on phosphodiesterase 2 (PDE2)/cAMP/PKA axis and the phosphorylation of mitochondrial transcription factor A (TFAM) as well as the growth of CRC cells were systematically investigated both *in vitro* and *in vivo.* Our findings demonstrated that MCU-induced mitochondrial Ca^2+^ uptake activated mitochondrial PDE2 in CRC cells. Moreover, overexpression MCU in CRC led to a 1.9-fold increase in Ca^2+^ uptake compared to control cells. However, knockdown of MCU resulted in 1.5-fould decrease in Ca^2+^ uptake in mitochondria compared to the controls. Activation of mitochondrial PDE2 significantly inhibited the activity of mitochondrial protein kinase A (PKA), which subsequently leads to decreased phosphorylation of TFAM. Our data further revealed that PKA regulates the phosphorylation of TFAM and promotes the degradation of phosphorylated TFAM. Thus, TFAM protein levels accumulated in mitochondria when the activity of PKA was inhibited. Overall, this study showed that the overexpression of MCU enhanced CRC growth through promoting the accumulation of TFAM proteins in mitochondria. Conversely, knockdown of MCU in CRC cells resulted in decreased CRC growth. Collectively, these data suggest that the mitochondrial Ca^2+^-activated PDE2/cAMP/PKA axis plays a key role in regulating TFAM stability and the growth of CRC cells.

## Introduction

Colorectal cancer (CRC) is one of the most deadly and commonly diagnosed cancers worldwide (1). Moreover, the incidence of CRC is steadily rising, especially in developing countries that are adopting the “western” way of life (1, 2). Although substantial progress has been achieved in the treatment of CRC, the mortality of CRC is still high. Therefore, the mechanism underlying the progression of CRC needs to be explored urgently.

Mitochondria harbor a Ca^2+^ buffering system and thus play a critical role in regulating intracellular calcium homeostasis (3). Over the past decade, a growing number of studies have shown that dysregulation of mitochondrial calcium homeostasis is closely linked with the progression of various types of cancers (4, 5). For example, our and other groups have demonstrated that mitochondrial calcium uniporter (MCU), a key mediator of mitochondrial Ca^2+^ uptake, is upregulated in several cancer cell types, including CRC (6), breast cancer (7), hepatocellular carcinoma (HCC) (8) and glioblastoma (GBM) (9). Moreover, a series of studies clearly indicate that the suppression of the mitochondrial Ca^2+^ uptake by the MCU inhibitor causes proliferation arrest, compromised migration, and cell death in several types of cancer cells (10, 11). Among them, our recent study has demonstrated that upregulated MCU enhances mitochondrial calcium uptake to promote mitochondrial biogenesis and colorectal cancer growth by suppressing phosphorylation of mitochondrial transcription factor A (TFAM) (6). However, the mechanism underlying the inhibition of TFAM phosphorylation by MCU-mediated mitochondrial Ca^2+^ uptake remains to be explored.

TFAM is a key regulator of mitochondrial DNA (mtDNA) replication and mitochondrial biogenesis (12). Altered mtDNA replication and mitochondrial biogenesis are closely associated with cancer cell proliferation and metastasis (13, 14). Thus, deregulation of TFAM has been implicated in various types of cancer, including CRC. For instance, TFAM is found to be aberrantly expressed in CRC cells and a high TFAM expression can serve as a useful marker for tumor progression in CRC patients (15). Because the fact that TFAM plays a significant role in carcinogenesis, the expression of TFAM is strictly controlled in cells. A study suggests that TFAM protein levels is regulated by phosphorylation (16). A detailed investigation revealed that TFAM is phosphorylated by cAMP-dependent protein kinase A (PKA) and that phosphorylated TFAM is degraded by Lon protease (16). Moreover, another study revealed that prune, localized in the mitochondrial matrix, is responsible for stabilizing TFAM by inhibiting the mitochondrial cAMP/PKA signaling pathway (17). A growing number of studies have indicated that PDE2, which is responsible for hydrolyzing the phosphodiester bond in the second messenger molecule cyclic adenosine monophosphate (cAMP) and cyclic guanosine monophosphate (cGMP), is instrumental for the growth and invasion of human malignant melanoma cells and osteosarcoma cells (18, 19). Although the significance of PDE2 in carcinogenesis has been investigated, the biological function of PDE2/cAMP/PKA axis-mediated TFAM stability in CRC growth remains to be explored.

In the present study, the regulatory effects of mitochondrial Ca^2+^ on mitochondrial PDE2/cAMP/PKA axis and the phosphorylation of TFAM, as well as the growth of colorectal cancer cells with MCU overexpression and knockdown were systematically investigated both *in vitro* and *in vivo*.

## Materials and Methods

### Cell Line and Animal Model

The normal human colorectal epithelial cell line FHC and CRC cell lines T84, SW1116, LS174T, HCT116 and RKO were purchased from the American Type Culture Collection. Cells were cultured in RPMI-1640 medium or DMEM supplemented with 10% fetal bovine serum and 1% penicillin/streptomycin solution.

LS174T cells with MCU knockdown or MCU overexpression were injected into five-week-old male Balb/c nude mice (six per group) to generate *in vivo* subcutaneous xenograft models. Tumor volume was measured every three days for 30 days and mice were sacrificed to measure of the wet weight of the excised tumors. All animal experiments were performed in compliance with the policies of the Institutional Animal Care and Use Committee of the Fourth Military Medical University (Permission number: IACUC-20170105; date issued: 2017-01-01).

### Antibodies and Reagents

The primary antibodies and their working concentrations used in this study are listed in **Supplementary Material Table 1.2.3**. The Mitochondria Fractionation Kit (Cat# C3601) was purchased from Beyotime (Biotechnology, China). PDE2 Activity Assay Kit (Cat# ab139460), PKA Kinase Activity Assay Kit (Cat# ab139435), cAMP Assay (Cat# ab65355) and ODQ (Cat# ab120022) were all purchased from Abcam (Abcam, United Kingdom). Go 6983 (Cas# 133053-19-7) inhibitor was from MCE (MCE, USA). The CellTiter 96^®^ Aqueous One Solution Cell Proliferation Assay was purchased from Promega (Cat# G3581, Promega, USA) and 5-ethynyl-2’-deoxyuridine (EdU)-incorporation assay kit was purchased from Ribobio (Cat# C10310, Ribobio, China). Bay 60-7550 (Cat# HY-14992) and H89 (Cat# HY-15979) were purchased from Med Chemexpress (Med Chemexpress, USA).

### Overexpression and Knockdown of Target Genes

To overexpress human MCU, PDE2A and TFAM proteins, the cDNA extracted from LS174T cells was used as a template to amplify the *MCU*, *PDE2A* and *TFAM* genes the primers listed in **Supplementary Material**. The PCR-amplified target genes were then cloned into the pcDNA™ 3.1 (+) vector (Invitrogen, USA). To generate shRNA expression vectors, a small hairpin RNA (shRNA) containing sequences targeting the human PDE2A, PKA or MCU was cloned into the pSilencer™ 3.1 puro vector (Ambion, USA). The shRNA control vector was generated by cloning a control shRNA into the pSilencer™ 3.1 puro vector. Cells were transfected with siRNA and plasmids using Lipofectamine 2000 transfection reagent (Invitrogen, USA) following the protocols recommended by the manufacturer. The siRNA sequences were synthesized (GenePharma, China) and are listed in **Supplementary Material**.

### Western Blot Analysis

Western blot experiments were carried out using the standard protocol described previously (20). Briefly, cells were lysed using RIPA buffer (Xi’an JingCai Biotechnology Company, China) with addition of protease and phosphatase inhibitors (Roche, Switzerland). The SDS-polyacrylamide gel was utilized to separate the target protein from the total proteins extracted from the lysed cells, followed by transferring the separated proteins to a PVDF membrane. The membrane was incubated with the primary antibody at 4°C overnight and then probed with a horseradish peroxidase-conjugated secondary antibody for 2h at room temperature. An enhanced chemiluminescence system was employed to detect the protein. Phosphorylated TFAM was detected using a previously described method (6). The band intensity in Western blot analyses was analyzed by Image J software (NIH, Bethesda, MD) and represented as the fold change of corresponding control value. The dilutions of antibodies are listed in **Supplementary Material**.

### Preparation of Mitochondrial Subcompartments and Proteinase K Protection Assays

The Mitochondria Fractionation Kit for Cultured Cells (Beyotime Biotechnology Cat# ab139460, China) was employed to isolate the mitochondria mainly according to the manufactures’ protocols. Firstly, 2 × 10^7^ cells were collected and centrifuged at 1000× rcf for 5 min. Secondly, Mitochondria Isolation Reagents were used to isolate mitochondria according to the manufactures’ protocols. All operations were performed on ice. Mitochondrial matrix was obtained by removing its outer membrane following the method reported previously (21). Briefly, isolated mitochondria were washed and suspended in STE containing 0.1% (w/v) fat-free BSA. The mitochondria were diluted with STE/BSA to a final concentration of 50 mg.ml-1 and stirred on ice. 0.5 ml of digitonin (6.25 mg.ml-1) was added dropwise over 2 min and the solution was kept on ice for a further 13 min. Three volumes of STE/BSA were added and the mixture was homogenized 4 times with a tight homogenizer. The mitochondria matrix fraction was collected by centrifugation for 10 min at 10,000 g.

The protease K protection assays were performed according to the method published previously (22). Briefly, 20μg/ml protease K (PK) were added into 20μg mitochondria protein for 20 minutes on ice. Then PK was inactivated with 2mM phenylmethanesulfonyl fluoride (PMSF) for min on ice. The mitochondria was solubilized with 1% Triton X-100 for 15 min on ice prior to PK treatment.

### Drug Administration

Bay 60-7550 (Cat# HY-14992, Med Chemexpress, USA), a PDE2 specific inhibitor, was employed in this study. Bay 60-7550 was dissolved in 1% dimethyl sulfoxide (DMSO), freshly prepared before administration. Mice under diethyl ether anesthesia received a single dose of Bay 60-7550 (1mg/kg) through intraperitoneal injection. The Bay 60-7550 inhibitor was injected into mice every other day for 30 days.

### Measurement of Mitochondrial Ca^2+^

The amount of mitochondrial Ca^2+^ was measured following the experimental protocol described previously (6). In brief, the plasmid containing the mitochondrial matrix-targeted fluorescent tagged inverse pericam was used to transfect CRC cells. The cells were then visualized under a confocal laser scanning microscope FV1000 (Olympus Corporation, Japan). Histamine (10µM) was added to trigger mitochondrial Ca^2+^ uptake after 30 s of baseline recording. Confocal images were recorded every 10 s at 545 nm excitation. All instrument settings (laser power, confocal slit opening, spectral bandwidth pixel excitation time, and photomultiplier sensitivity) were identical with control cells using custom-made software.

### Activity Assays of Mitochondrial PDE2 and PKA

The enzymatic activities of mitochondrial PDE2 and PKA were determined with the PDE2 Activity Assay Kit and PKA Kinase Activity Assay Kit (Abcam, United Kingdom), respectively, following the manufacturers’ protocols. Briefly, cells were harvested in a 15 mL conical tube (1 x 10^7^/mL). The cells were lysed with the reagent in the kit and subsequently the protein concentration was measured. Then the enzymatic activities of mitochondrial PDE2 and PKA were assayed using the reagent kit according to the manufacturers’ protocols. All values were normalized to total cellular protein concentration.

### Immunohistochemical (IHC) Staining

Immunohistochemical (IHC) experiments were carried out following the experimental procedure described previously (20). In brief, sections were deparaffinized and hydrated. Antigen was retrieved by treating with hot citrate buffer (pH = 6.0) under pressure. Then, sections were incubated with primary antibody overnight at 4°C. Color was developed using DAB substrate followed by hematoxylin counterstaining. The expression levels in terms of IHC score were independently assessed by two pathologists according to the intensity and percentage of positive cells.

### Cell Viability and Cell Proliferation Assays

Cell viability and cell proliferation assays were carried out using the methods described methods (23). Briefly, the proliferation of CRC cells and non-cancerous colon cells were measured using the CellTiter 96^®^ Aqueous One Solution Cell Proliferation Assay (Promega, USA) according to the manufacturers’ instructions. CRC cells and non-cancerous colon cells were seeded with 2000 cells on a 96-well plate and incubated for 4 days. Cell proliferation was detected daily based on the absorbance at 490 nm. To assess cell proliferation, the 5-ethynyl-2’-deoxyuridine (EdU)-incorporation assay kit (Ribobio, China) was used according to manufactures’ protocols.

### Site-Directed Mutagenesis

Plasmid constructs expressing mutant TFAM proteins including TFAM^S55D^ and TFAM^S55A^, which are also referred to as pcDNA-TFAM^S55D^ and pcDNA-TFAM^S55A^, were generated previously in our lab (6).

### Statistical Analysis

SPSS 17.0 software (SPSS. Inc., USA) was utilized to analyze the data. Values are shown as the mean ± SEM from three independent experiments, where appropriate. Student’s t-test was employed to analyze significant differences between two groups or multiple groups. A *P-Value <*0.05 was considered statistically significant.

## Results

### MCU-Mediated Mitochondrial Ca^2+^ Uptake Activates Mitochondrial PDE2 to Inhibit PKA Activity

**Figure 1:**
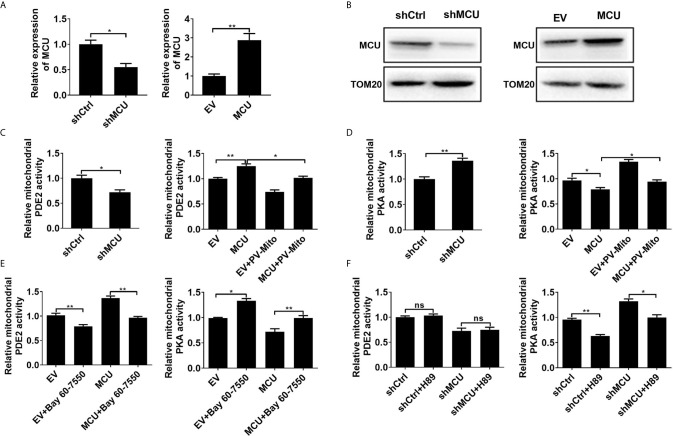
MCU-mediated mitochondrial Ca2+ uptake activates mitochondrial PDE2 to inhibit mitochondrial PKA activity. **(A)** QRT-PCR analysis for the mRNA level in LS174T cells treated as indicated. **(B)** Western blot analysis of protein level of mitochondrial MCU in LS174T cells treated as indicated. **(C)** Relative mitochondrial PDE2 activity in LS174T cells treated as indicated. **(D)** Relative mitochondrial PKA activity in LS174T cells treated as indicated. **(E)** Relative activities of mitochondrial PDE2 and PKA in LS174T cells treated as indicated (Bay 60-7550, 4.7nM PDE2 inhibitor for 24h). **(F)** Relative activities of mitochondrial PDE2 and PKA in LS174T cells treated as indicated (H89, 1μM PKA inhibitor for 24h). MCU, expression vector encoding mitochondrial calcium uniporter; shCtrl, expression vector encoding control shRNA; shMCU, expression vector encoding shRNA against MCU; EV, empty vector; PV-Mito, expression vector encoding parvalbumin with mitochondria target sequence. *P < 0.05; **P < 0.01; ns, not significant.

To explore whether MCU-mediated mitochondrial Ca^2+^ uptake was involved in regulating the activity of mitochondrial PDE2 and PKA, MCU was overexpressed or knocked down in CRC cells. We first determined the expression level of MCU in one normal human colorectal epithelial cell line (FHC) and a series of CRC cell lines including T84, SW116, LS174T, HCT116 and RKO. The qRT-PCR and Western blot analysis revealed that the expression level of MCU is significantly higher in CRC cell lines compared to normal colorectal epithelial cell line (**Supplementary Material Figures 1A, B**). To further explore the biological functions of MCU in CRC cells, the expression of MCU was knocked down or over-expressed in LS174T cells which had relatively high levels of MCU expression. As shown in **Figures 1A, B**, successful knockdown and overexpression of mitochondrial MCU at both mRNA and protein levels were confirmed. As expected, the data showed that the resting matrix Ca^2+^ level was lower in CRC cells with MCU knockdown compared to the controls, whereas the resting matrix Ca^2+^ level was remarkably higher in MCU-overexpressed CRC cells compared to the controls (**Supplementary Material Figure 1C**). Moreover, the overexpression of MCU in CRC cells markedly enhanced mitochondrial Ca^2+^ uptake, while knockdown of MCU decreased mitochondrial Ca^2+^ uptake (**Supplementary Material Figure 1D**). These two experimental groups showed statistically significant difference. Overexpression MCU in CRC led to a 1.9-fold increase in Ca^2+^ uptake compared to control cells. However, knockdown of MCU resulted in 1.5-fould decrease in Ca^2+^ uptake in mitochondria compared to the controls. Then mitochondria were isolated from MCU-overexpressing and MCU-knockdown CRC cells. The knockdown of MCU alone or in combination with PDE2 overexpression were confirmed by Western blot (**Figure S1E**). Moreover, the overexpression of MCU alone or in combination with PDE2 inhibition were validated by Western blot as well (**Figures S1F, G**).

Then the enzymatic activity of PDE2 and PKA in mitochondria was measured. As shown in **Figures 1C, D**, the knockdown of MCU resulted in decreased mitochondrial PDE2 activity and increased mitochondrial PKA activity in CRC cells compared with the control group. However, overexpression of MCU exhibited the opposite effect. The [Ca^2+^]_m_ buffering by PV-Mito significantly reversed the effects caused by MCU overexpression. Moreover, we found that the PDE2 inhibitor BAY60-7550 remarkably increased the activity of mitochondrial PKA by inhibiting PDE2 activity in CRC cells with or without MCU overexpression compared with the corresponding controls (**Figure 1E**). In contrast, the PKA inhibitor H89 only inhibited the activity of mitochondrial PKA, but had no effect on the activity of mitochondrial PDE2 in CRC cells (**Figure 1F**). These results indicate that MCU-mediated mitochondrial Ca^2+^ uptake increases the activity of mitochondrial PDE2 and subsequently inhibits the activity of mitochondrial PKA in CRC cells.

Due to the low expression of MCU in FHC cells, we measured the activity of PDE2 and PKA in a non-cancerous colon cell line FHC with MCU overexpression. We found that these results in FHC cells are similar to those obtained in CRC cells treated with MCU overexpression (**Supplementary Material Figures 2A–D**). Given that overexpression or down-regulation of MCU impinges on mitochondrial PDE2 activity. We measured the level of cAMP in isolated mitochondria from CRC cells with MCU overexpression and knockdown. Our data indicated that the knockdown of MCU resulted in an increase of the levels of cAMP in CRC cells compared with the control group, whereas the overexpression of MCU exhibited the opposite effect (**Supplementary Material Figure 2E**). Furthermore, we used the phospho-PKA substrate antibody to directly assess the mitochondrial PKA activity in the mitochondria isolated from CRC cells treated with MCU overexpression. The data showed that the phosphorylation level of PKA substrates was decreased in CRC cells with MCU overexpression. Conversely, the addition of PV-Mito and bay 60-7550 significantly decreased the phosphorylation level of PKA substrates (**Supplementary Material Figures 2F, G**).

Previous studies showed that PDE2 is a cGMP-activated PDE2, degrading both cGMP and cAMP (24). Moreover, it has been reported that Ca^2+^ can activate the soluble guanylate cyclase (sGC) through GCAP1(Guanylyl Cyclase Activator Protein 1) and GCAP2(Guanylyl Cyclase Activator Protein 2), and this, through cGMP production, could activate PDE2 (24). We used of siRNA of GCAP1 and inhibitors of sGC to verify this hypothesis. Western blot analysis showed that the protein level of PDE2A was not obviously changed when the expression of GCAP1 was inhibited in MCU-overexpressed CRC cells. However, the activity of PDE2 was significantly inhibited in CRC cells (**Supplementary Material Figures 3A, B**). Furthermore, it has been reported that PDE2 could also be activated by protein kinase C (PKC) (25). We tested this hypothesis by adding PKC inhibitor into CRC cells with MCU downregulation. Our data indicated that the addition of PKC inhibitor did not cause a change in mitochondrial PDE2 activity, suggesting that Ca^2+^ may regulate the activity of PDE2 through GCAP1 (**Supplementary Material Figures 3A, B**).

### PDE2-Mediated Inhibition of PKA Activity Is Essential for Mitochondrial Ca^2+^ Induced Dephosphorylation of TFAM

**Figure 2:**
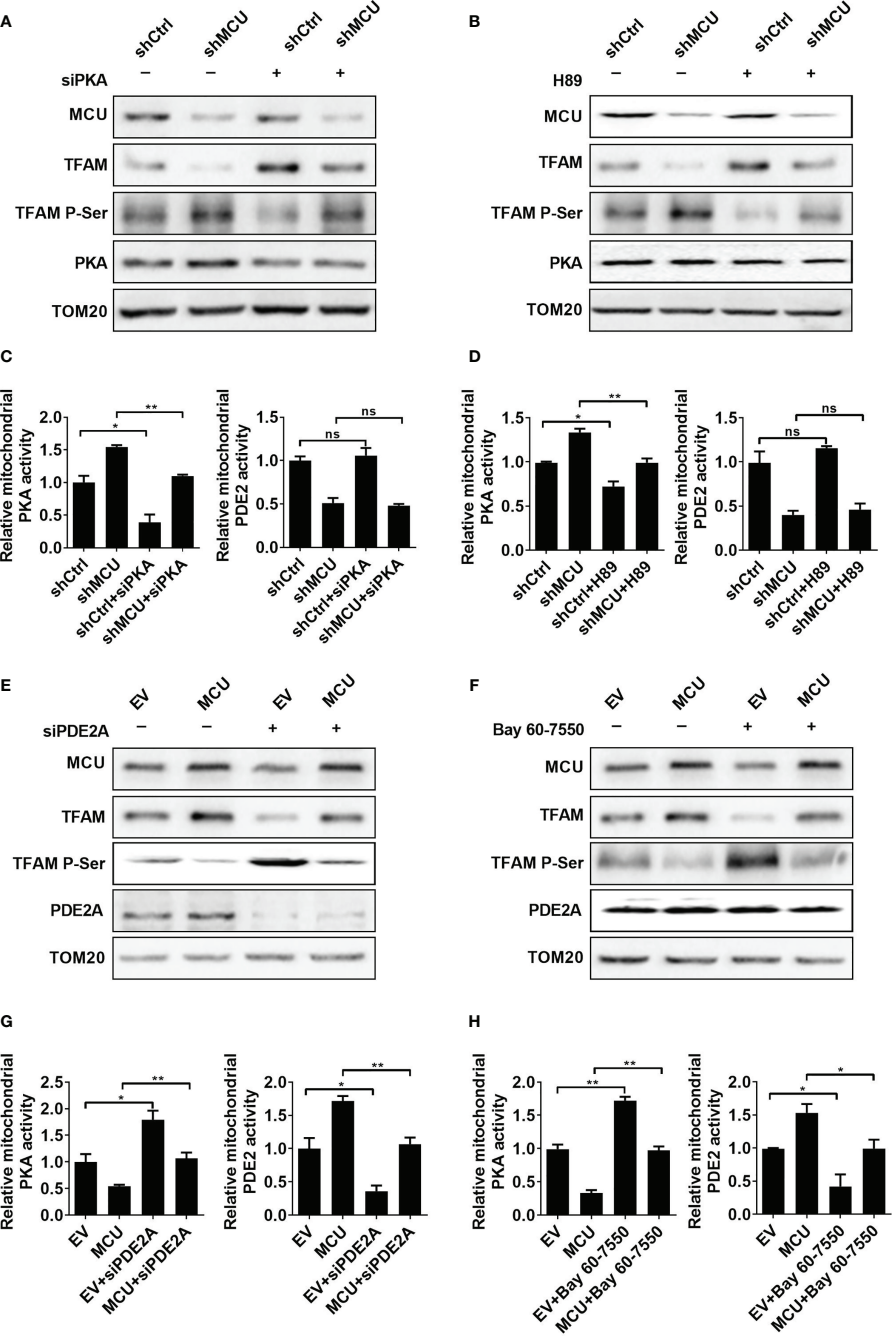
PDE2-mediated inhibition of PKA activity is essential for mitochondrial Ca2+ induced dephosphorylation of TFAM. **(A, B)** Western blot analysis for protein levels of mitochondrial MCU and PKA, TFAM and phosphorylated TFAM in LS174T cells treated as indicated. siPKA: siRNA against PKA. PKA inhibitor H89 (1 μM) was used. **(C, D)** Relative activities of mitochondrial PDE2 and PKA in LS174T cells treated as indicated. **(E, F)** Western blot analysis for protein levels of mitochondrial MCU, PDE2, TFAM and phosphorylated TFAM in LS174 cells treated as indicated. **(G, H)** Relative activities of mitochondrial PDE2 and PKA in LS174T cells treated as indicated. siPDE2A: siRNA against PDE2A. PDE2 inhibitor Bay 60-7550 (4.7 nM) was used. *P < 0.05; **P < 0.01; ns, not significant.

Considering that our previous study demonstrated the regulation of mitochondrial Ca^2+^ in TFAM phosphorylation, which is essential for controlling TFAM stability (6), we examined whether the PDE2/cAMP/PKA axis is engaged in regulating TFAM phosphorylation in human CRC cells. Our data showed that the decreased protein level of TFAM and increased TFAM phosphorylation, which were mediated by MCU knockdown, were significantly reversed when the activity of PKA was suppressed by siRNA or a PKA inhibitor, H89 (**Figures 2A, B**). Furthermore, the protein level and phosphorylation level of TFAM shown in **Figures 2A**, **B** had been quantified using the ImageJ software (**Supplementary Material Figures 4A, B**). Our data suggested that phosphorylation of TFAM leads to its degradation, which is consistent with previous studies (6, 16). In the meantime, we also examined the activities of mitochondrial PKA and PDE2 in MCU knocked-down CRC cells with addition of siPKA or H89. We found that the activity of mitochondrial PKA was restored to normal in CRC cells treated with MCU knockdown in combination with siPKA or H89 (**Figures 2C, D**). Similar results were obtained when the phospho-PKA substrate antibody was used to determine the PKA activity (**Supplementary Material Figure 4C**). However, the activity of PDE2 displayed no remarkable change, irrespective of the change of PKA (**Figures 2C, D**).

Furthermore, the inhibition of PDE2 activity either by siRNA or a PDE2 inhibitor Bay 60-7550 notably restored the phosphorylation of TFAM and then resulted in downregulation of TFAM in MCU-overexpressing CRC cells compared with controls (**Figures 2E, F**). The protein expression level and phosphorylation level of TFAM shown in **Figures 2E**, **F** had been quantified using the ImageJ software (**Supplementary Material Figures 4D, E**). Overexpression of MCU in CRC cells led to an increase in the activity of mitochondrial PDE2, whereas the activity of mitochondrial PKA was decreased compared with the control group. Similar results were obtained when the phospho-PKA substrate antibody was used to determine the PKA activity (**Supplementary Material Figure 4F**). However, the addition of siPDE2A or Bay 60-7550 significantly reversed the effect caused by MCU overexpression in CRC cells (**Figures 2G, H**). Additionally, we determined the phosphorylation of TFAM in CRC cells treated with siPKA in combination with siPDE2A. Our data indicated that TFAM phosphorylation is dependent on PKA (**Supplementary Material Figure 4G**).

The mitochondrial outer membrane was removed from the isolated mitochondria and then the matrix proteins were examined by Western blot experiments to detect the expression of the PDE2A and PKA. Our data demonstrated the presence of PDE2A and PKA in mitochondrial matrix (**Supplementary Material Figure 4H**). Collectively, these findings provide supporting evidence that the PDE2/cAMP/PKA axis participates in MCU-mediated regulation of TFAM phosphorylation in CRC cells.

### Activation of PDE2 Is Essential for Mitochondrial Ca^2+^-Mediated CRC Cell Growth

**Figure 3:**
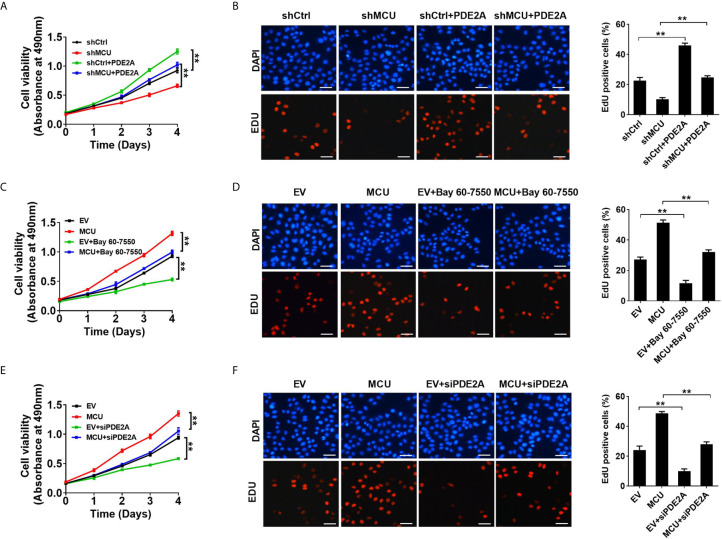
Activation of PDE2 is essential for mitochondrial Ca2+-mediated CRC cell proliferation *in vitro*. **(A, C, E)** MTS assay for cell viability and **(B, D, F)** EdU incorporation assays for cell proliferation in LS174T cells with treatments as indicated (scale bar, 20 μm). PDE2A, expression vector encoding PDE2A; siPDE2A, siRNA against PDE2A. **P < 0.01.

To explore the functional roles of PDE2-mediated decrease of TFAM phosphorylation in CRC cell growth, we first examined the effect of PDE2 activity on CRC growth *in vitro*. As shown in **Figures 3A, B**, the overexpression of PDE2 significantly promoted cell proliferation and increased the percentage of EDU-positive cells compared with the corresponding controls. In addition, the overexpression of PDE2 notably reversed the effect of MCU knockdown in CRC cells (**Figures 3A, B**). Consistently, inhibition of PDE2 activity by siRNA or inhibitor Bay 60-7550 significantly suppressed cell proliferation and decreased the percentage of EDU-positive cells (**Figures 3C–F**). Moreover, the inhibition of PDE2 activity clearly reversed the effect of MCU overexpression in CRC cells (**Figures 3C–F**). Meanwhile, we also explored the functional roles of PDE2-mediated decrease of TFAM phosphorylation in non-cancerous colon cell growth. The results showed that cell viability and cell proliferation were enhanced in normal human colorectal epithelial cells with MCU overexpression. In addition, inhibition of PDE2 activity by siPDE2A or PDE2 inhibitor significantly suppressed cell viability and decreased the percentage of EDU-positive cells in FHC cells (**Supplementary Material Figures 5A–D**).

**Figure 4:**
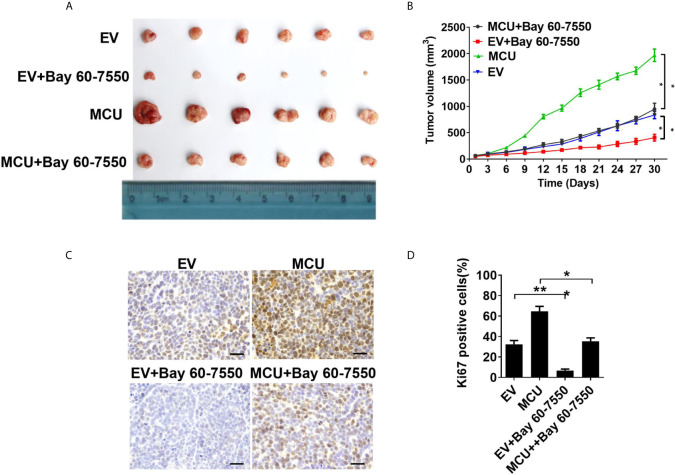
Activation of PDE2 is essential for mitochondrial Ca2+-mediated CRC cell proliferation *in vivo*. **(A)** Dissected tumors from sacrificed mice and **(B)** tumor growth curves of subcutaneous xenograft tumor developed from LS174T cells with treatments as indicated. **(C)** Representative IHC staining images of Ki67 and **(D)** percentage of Ki67-positive cells in xenograft tumor developed from LS174T cells with treatments as indicated (scale bar, 50 μm). *P < 0.05; **P < 0.01.

We further investigated the physiological role of PDE2-mediated decrease of TFAM phosphorylation in CRC growth *in vivo*. the xenograft nude mice models were established with CRC cells. Our data showed that treatment with the PDE2 inhibitor Bay 60-7550 markedly suppressed the growth of CRC xenografts and clearly reversed the effect of MCU overexpression on CRC growth (**Figures 4A, B**). Consistently, IHC analysis indicated that CRC xenograft treated with Bay 60-7550 had a lower percentage of Ki67-positive cells compared with the corresponding controls (**Figures 4C, D**). Collectively, these findings indicate that PDE2 harbors a vital role in mitochondrial Ca^2+^-regulated CRC growth both *in vitro* and *in vivo*.

### Decreased Phosphorylation of TFAM Is Involved in Mitochondrial Ca^2+^-Mediated CRC Growth

**Figure 5:**
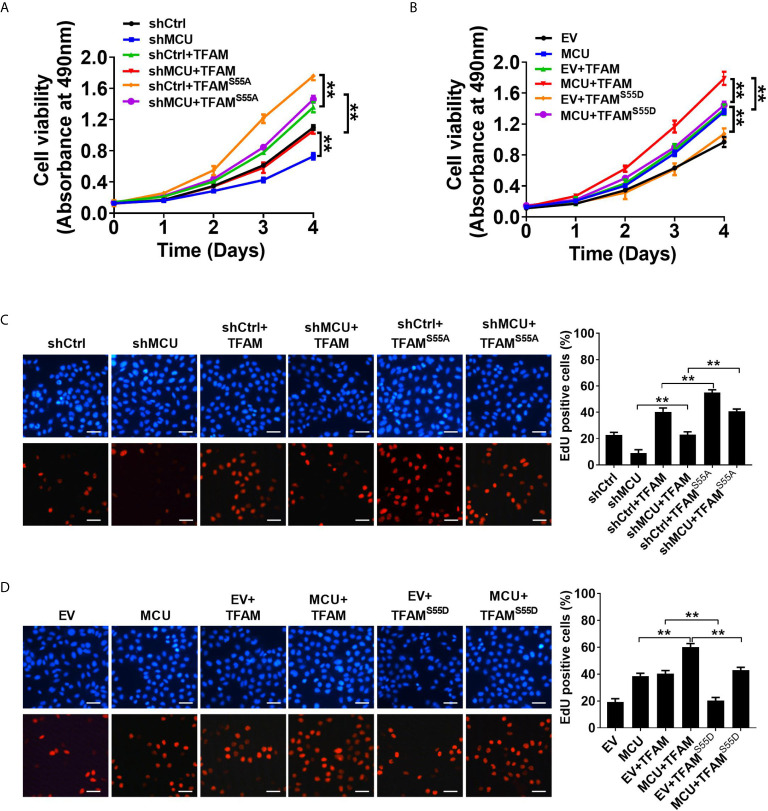
Decreased phosphorylation of TFAM is involved in mitochondrial Ca2+-mediated CRC growth *in vitro*. **(A, B)** MTS assay for cell viability and **(C, D)** EdU incorporation assays for cell proliferation in LS174T cells with treatments as indicated (scale bar, 20 μm). **P < 0.01.

**Figure 6:**
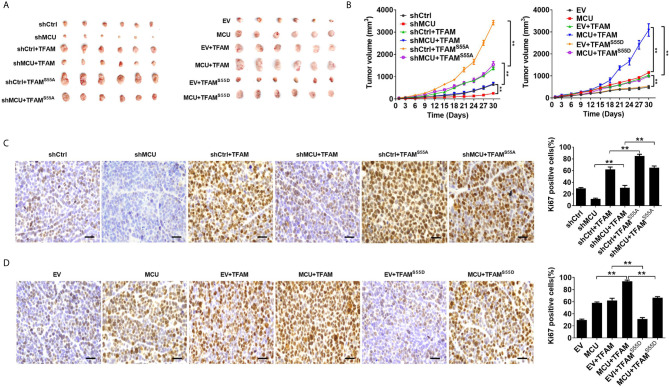
Decreased phosphorylation of TFAM is involved in mitochondrial Ca2+-mediated CRC growth *in vivo*. **(A)** Dissected tumors from sacrificed mice and **(B)** tumor growth curves of subcutaneous xenograft tumor developed from LS174T cells with treatments as indicated. **(C, D)** Representative IHC staining images of Ki67 and (shMCU and MCU group) percentage of Ki67-positive cells in xenograft tumor developed from LS174T cells with treatments as indicated (scale bar, 50 μm). ** P < 0.01.

We then sought to explore the effect of TFAM and its phosphorylation on MCU-regulated CRC growth *in vitro* and *in vivo*. As shown in **Figures 5A–D**, TFAM overexpression promoted cell proliferation and increased the rate of EDU-positive cells in CRC cells compared with the corresponding controls. A previous study revealed that TFAM was phosphorylated at serine-55 (23). Thus, the mutation of serine-55 to alanine (TFAM^S55A^) abolished its PKA-dependent phosphorylation, whereas the mutation of serine-55 to aspartic acid (TFAM^S55D^) mimics the sustained phosphorylation of TFAM (26). Furthermore, we found that the overexpression of TFAM^S55D^ resulted in a lower cell proliferation and EDU-positive cell percentage in CRC cells compared with cells overexpressed with wild type (WT) TFAM. On the contrary, the overexpression of TFAM^S55A^, which inhibits TFAM phosphorylation, exhibited the opposite effect on cell growth in CRC cells (**Figures 5A–D**). Consistent with the *in vitro* experiments, similar results were obtained in *in vivo* CRC xenograft nude mice models (**Figures 6A–D**). These data implied that TFAM overexpression resulted in a faster growth and higher percentage of Ki67-positive cells in CRC xenografts compared with the corresponding controls. Conversely, we found that the overexpression of TFAM^S55D^ led to a slower growth and lower Ki67-positive cell percentage in CRC xenografts than in those overexpressing WT TFAM. Moreover, the overexpression of TFAM^S55A^ exhibited the opposite effect (**Figures 6A–D**). To sum up, these data support the notion that TFAM phosphorylation plays a vital role in MCU-regulated CRC growth.

## Discussion

In this study, our results demonstrated that MCU-induced mitochondrial Ca^2+^ uptake significantly enhanced the activity of the mitochondrial PDE2/cAMP/PKA axis, which subsequently led to decreased phosphorylation of TFAM and thus accumulation of TFAM. Consistently, a previous study demonstrated that a phosphodiesterase prune, a cAMP-degrading enzyme, stabilizes TFAM to promote mtDNA replication by inhibiting mitochondrial cAMP/PKA signaling (17). Moreover, our results indicated that both PDE2 and TFAM play critical roles in mitochondrial Ca^2+^-induced CRC growth.

It has been well established that Ca^2+^ is critical for activating several family members of PDEs. For instance, studies have shown that the enzymatic activity of PDE1 and the localization of PDE4 are mediated by an increase in [Ca^2+^]*_i_* (27, 28). Our findings establish that MCU-mediated Ca^2+^ uptake plays essential roles in regulating mitochondrial PDE2, which is known to possess dual substrate specificity for cAMP and cGMP (29). Thus, PDEs are considered to play vital roles in modulating the cross-talk between cAMP and Ca^2+^ signaling (27). Moreover, it has been suggested that cAMP harbors a significant role in activating PKA, which in turn regulates diverse physiological processes (30, 31).

Further investigation revealed that activation of the PDE2/cAMP/PKA axis by Ca^2+^ possesses a key role in regulating the stability of TFAM. Our data imply that downregulation of mitochondrial PKA activity mediated by mitochondrial PDE2 resulted in a decrease in the phosphorylation of TFAM, which then leads to accumulation of TFAM protein in mitochondria. These findings are consistent with a previous report, which suggests that PKA-mediated TFAM phosphorylation is crucial for the stability of TFAM (16). The overexpression and/or knockdown of mitochondrial MCU, TFAM, TFAM^S55A^ and TFAM^S55D^ in LS174T cells were confirmed by western blot (**Supplementary Material Figures 6A, B**). Our data further showed that phosphorylation of TFAM led to its degradation, whereas inhibiting the phosphorylation of TFAM resulted in its accumulation in mitochondria.

Moreover, compartmentalized mitochondrial cAMP signaling mediated by the PDE/PKA axis has been demonstrated to be important for TFAM stabilization (17), which is also consistent with the findings of the current study.

The biological functions of several PDE family members have been intensively studied in CRC. For instance, one study showed that cAMP/PDE4B signals possess an important role in mediating the malignant phenotype of CRC cells by regulating the mammalian target of rapamycin (mTOR) signaling (32). Furthermore, PDE4D has been demonstrated to play an essential role in modulating cAMP levels in DLD-1 CRC cells, which in turn mediates the expression of Myc in CRC cells (33). Similarly, in the present study, we demonstrated that mitochondrial PDE2 also harbor a key role in promoting CRC growth by mediating the phosphorylation of TFAM. Furthermore, Monterisi et al. indicated that the cAMP/PKA signaling domain is localized at mitochondrial membranes and regulated by PDE2 (34), which is in agreement with our findings. Detailed investigations revealed that PDE2 mediates mitochondrial morphology and apoptotic cell death through local regulation of cAMP/PKA signaling (34). It has also been reported that erythro-9-(2-hydroxy-3-nonyl) adenine (EHNA), a PDE2 inhibitor, is capable of suppressing the growth of the human malignant melanoma PMP cell line (19). Collectively, these findings suggest that PDEs are important players in CRC.

Growing evidence indicates that TFAM is closely associated with CRC. For example, one study showed that the expression of TFAM is frequently upregulated in colon adenocarcinoma tissues compared with the corresponding paracancerous tissues (35). Additionally, a positive correlation between TFAM expression and TNM stage exists in CRC (15). Additionally, truncating mutation of TFAM is found to be frequently occurred in CRC, resulting in mtDNA depletion and cisplatin-induced apoptotic resistance of most microsatellite-unstable CRC (36). Collectively, these studies indicate that high TFAM expression can be a potential marker for tumor progression in CRC patients. Several studies also suggest that microRNAs, including microRNA-214 (miR-214) and microRNA-204 (miR-204), may play a pivotal in regulating the proliferation of CRC cells by mediating TFAM expression (37, 38). In the current study, our data uncovered a novel mechanism underlying TFAM-mediated CRC growth.

In conclusion, our findings uncover that MCU-induced mitochondrial calcium (Ca^2+^) uptake activated PDE2 in mitochondria, which in turn inhibited the activity of mitochondrial PKA. This subsequently led to decreased phosphorylation of TFAM and increased accumulation of TFAM in mitochondria. Taken together, our data indicate that the mitochondrial Ca^2+^-activated PDE2/cAMP/PKA axis is instrumental in regulating TFAM stability and the growth of CRC cells. These findings imply that targeting PDE2/cAMP/PKA axis-mediated TFAM accumulation could be a promising therapeutic strategy for CRC treatment.

## Data Availability Statement

The raw data supporting the conclusions of this article will be made available by the authors, without undue reservation.

## Ethics Statement

This study was performed with the approval from the Ethics Committee of Fourth Military Medical University (Permission number: KY20173189-1; Date issued: 2017-03-06). The patients/participants provided their written informed consent to participate in this study. All animal experiments were conducted according to the guidelines of the Institutional Animal Care and Use Committee of the Fourth Military Medical University (Permission number: IACUC-20170105; Date issued: 2017-01-01). Written informed consent was obtained from the owners for the participation of their animals in this study.

## Author Contributions

YZ, FZ, NW, ZZ, MJ, CJ, and JZhao performed the experiments and analyzed the data. YW, JZhang, and JX wrote the paper. TR, JX, XH, YW, and JZhao supervised and revised the paper. All authors contributed to the article and approved the submitted version.

## Funding

This study was supported by the China Postdoctoral Science Foundation (2020M683751, 2019M663985 and 2019M663984) and Basic Research Plan of Shanxi Natural Science (2021JQ-559). Key Research and Development Projects of Shanxi Province (2017SF-188), Natural Science Foundation of Shaanxi Provincial Department of Education (JM8020) and the National Natural Science Foundation of China (81872302, 82002511, 81802345, 81672340 and 82072722).

## Conflict of Interest

The authors declare that the research was conducted in the absence of any commercial or financial relationships that could be construed as a potential conflict of interest.
