# Supplementary material for: PDE2 Inhibits PKA-Mediated Phosphorylation of TFAM to Promote Mitochondrial Ca2+-Induced Colorectal Cancer Growth
Source: Front Oncol. 2021 Jun 21;11:663778. doi: 10.3389/fonc.2021.663778 (PMC8256694; doi:10.3389/fonc.2021.663778)
Supplement: Supplementary file 1 [file DataSheet_1.docx]

Supplementary Material

## Supplementary Figures


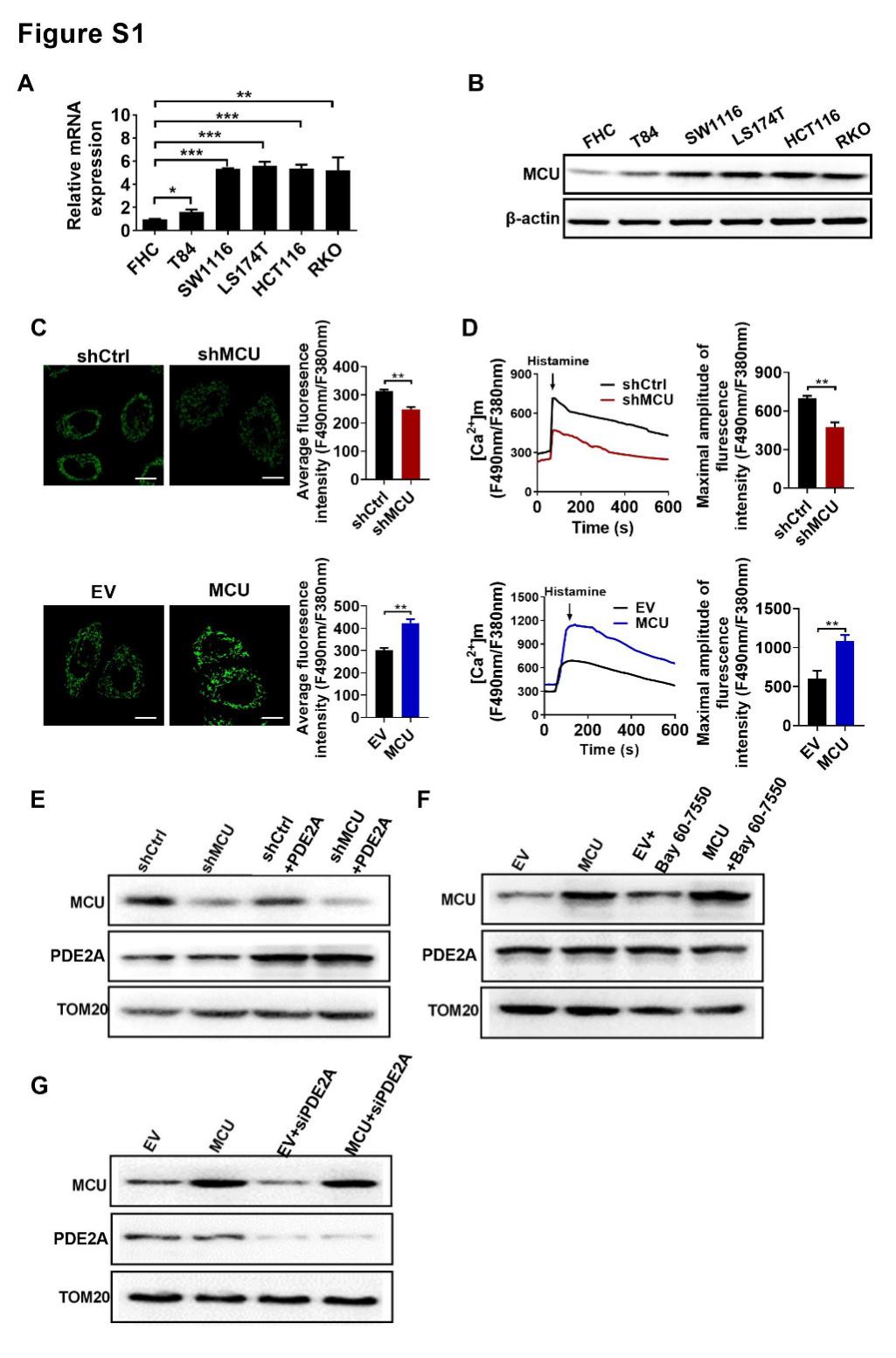


**Supplementary Figure 1. (A-B)** QRT-PCR and Western blot analysis for the mRNA and protein level of mitochondrial MCU, respectively, in CRC cell lines and normal human colorectal epithelial cell line. **(C)** Typical confocal microscope images of mitochondrial Ca^2+^ ([Ca^2+^]_m_) levels in LS174T cells treated as indicated. **(D)** Mitochondrial Ca^2+^ ([Ca^2+^]_m_) in LS174T cells treated as indicated. **(E-G)** Western blot analysis for protein levels of mitochondrial MCU and PDE2A in LS174T cells treated as indicated (shCtrl, control shRNA; shMCU, shRNA against MCU; MCU, expression vector encoding MCU; EV, empty vector; siPDE2A: siRNA against PDE2A). * *P<*0.05*;* ** *P<*0.01*.*


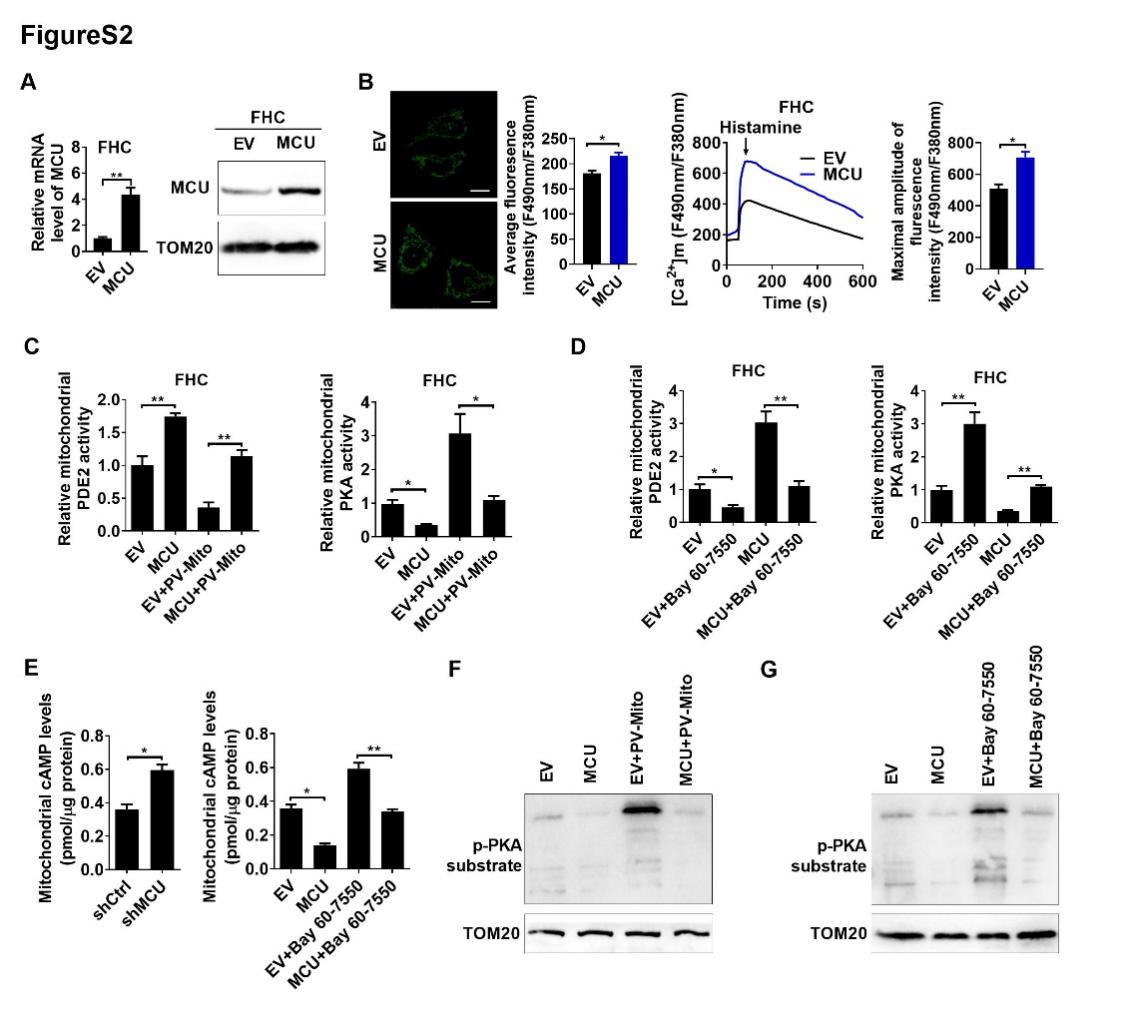


**Supplementary Figure 2. (A)** QRT-PCR and Western blot analysis for the mRNA and protein level of mitochondrial MCU, respectively, in FHC cells with treatments as indicated. **(B)** Mitochondrial Ca^2+^ ([Ca^2+^]_m_) in FHC cells treated as indicated. **(C and D)** Relative mitochondrial PDE2 and PKA activities in FHC cells treated as indicated. **(E)** The mitochondrial cAMP levels in LS174T cells treated as indicated. **(F-G)** Western blot analysis for the protein level of p-PKA substrate in LS174T cells with treatments as indicated. (shCtrl, control shRNA; shMCU, shRNA against MCU; MCU, expression vector encoding MCU; EV, empty vector; siPDE2A: siRNA against PDE2A; PV-Mito, expression vector encoding parvalbumin with mitochondria target sequence). * *P<*0.05*;* ** *P<*0.01*.*


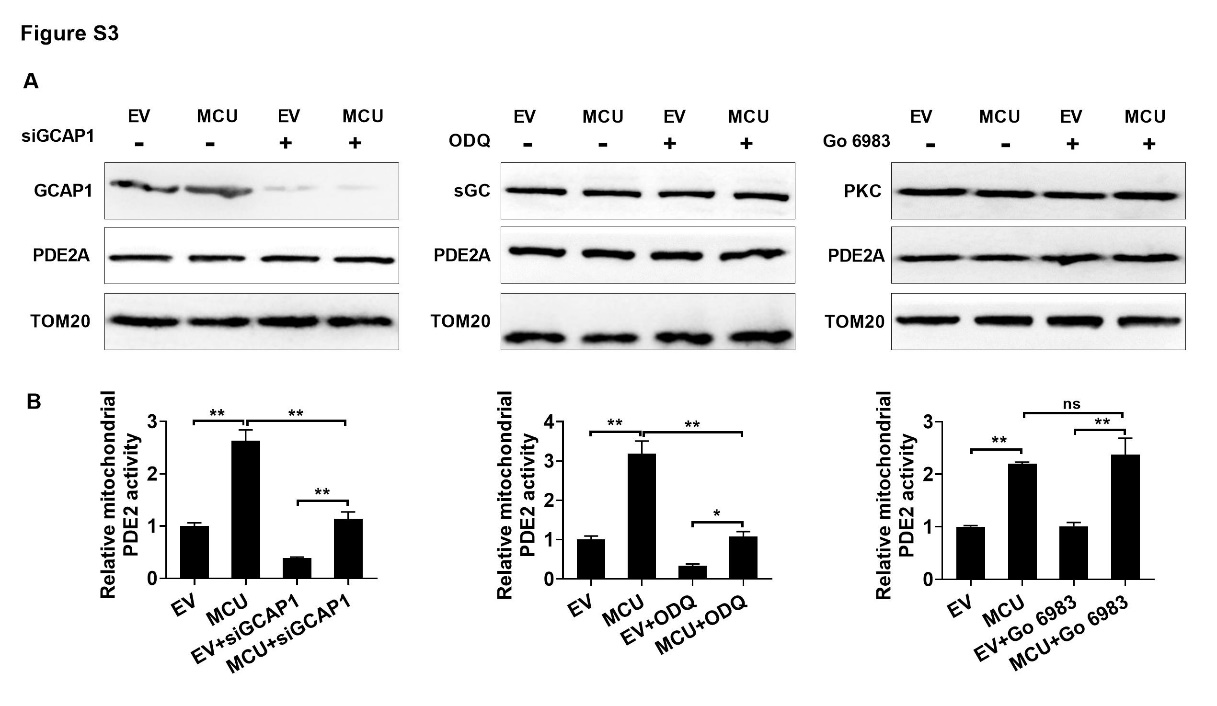


**Supplementary Figure 3**. **(A)**Western blot analysis for the protein level of mitochondrial GCAP1, sGC, PDE2A and PKC in LS174T cells with treatments as indicated. **(B)** Relative mitochondrial PDE2 activitiy in LS174T cells treated as indicated. (siCtrl, control siRNA; siGCAP1, siRNA against GCAP1; ODQ, sGC inhibitor (100 nM for 2h), PKC inhibitor (10 nM for 2h)). * *P<*0.05*;* ** *P<*0.01*.*


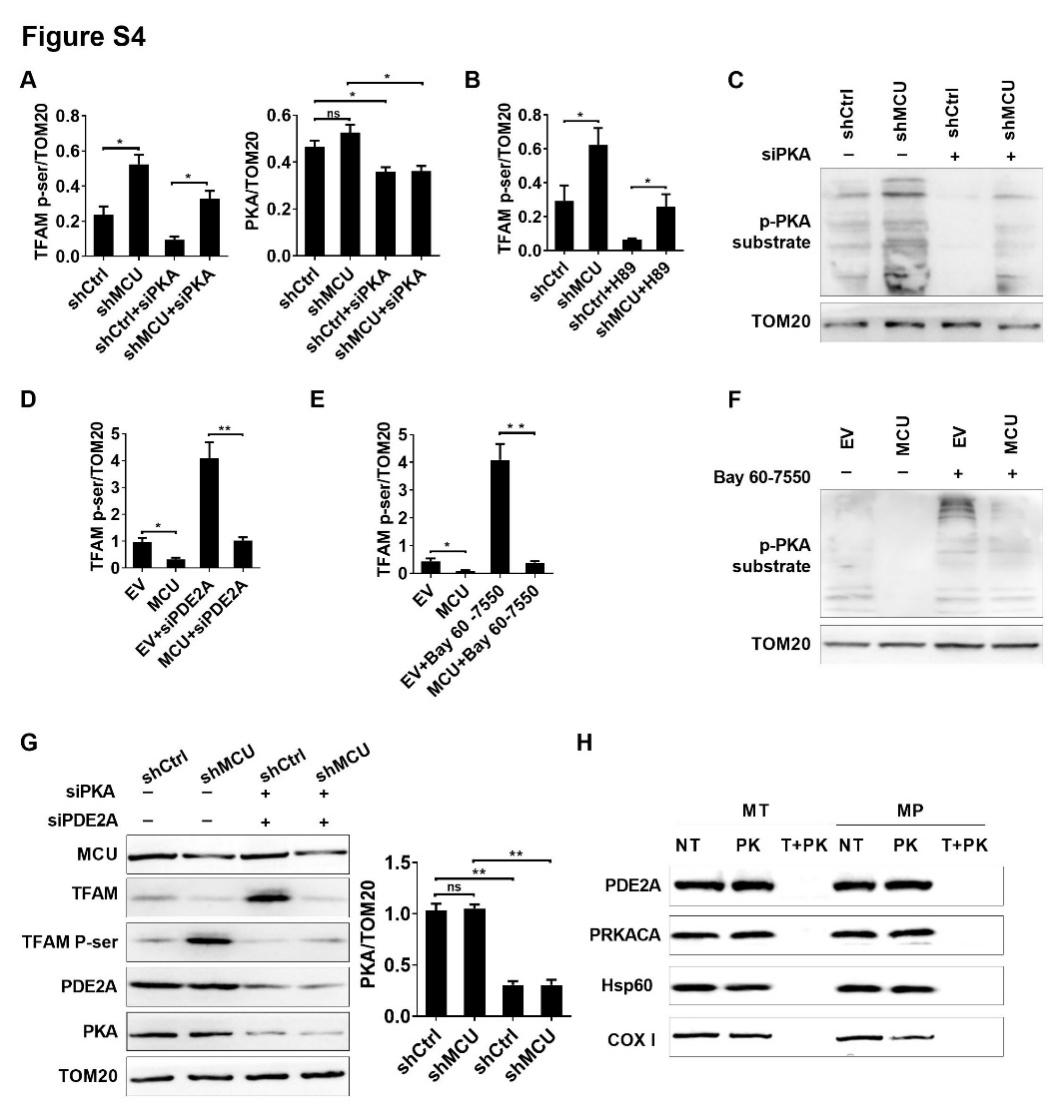


**Supplementary Figure 4**. **(A-B)** ImageJ analysis the protein levels of TFAM p-ser/TFAM and PKA. **(C)** Western blot analysis for the protein level of p-PKA substrate in LS174T cells with treatments as indicated. **(D-E)** ImageJ analysis the protein level of TFAM p-ser/TFAM. **(F)** Western blot analysis for the protein level of p-PKA substrate in LS174T cells with treatments as indicated. **(G)** ImageJ analysis the protein level of PKA and western blot analysis for protein levels of mitochondrial MCU, PKA and TFAM and phosphorylated TFAM in LS174T cells treated as indicated. siPKA: siRNA against PKA; siPDE2A: siRNA against PDE2A. **(H)** Western blot analysis for protein levels of PRKACA and PDE2A in mitochondria (MT) and mitochondrial matrix with inner membrane (MP) with treatments as indicated. NT, non-treated; PK, proteinase K; T+PK, Triton plus proteinase K. Hsp60, marker for mitochondrial matrix proteins; COX Ⅰ, marker for mitochondrial inner membrane proteins. * *P<*0.05*;* ** *P<*0.01*.*


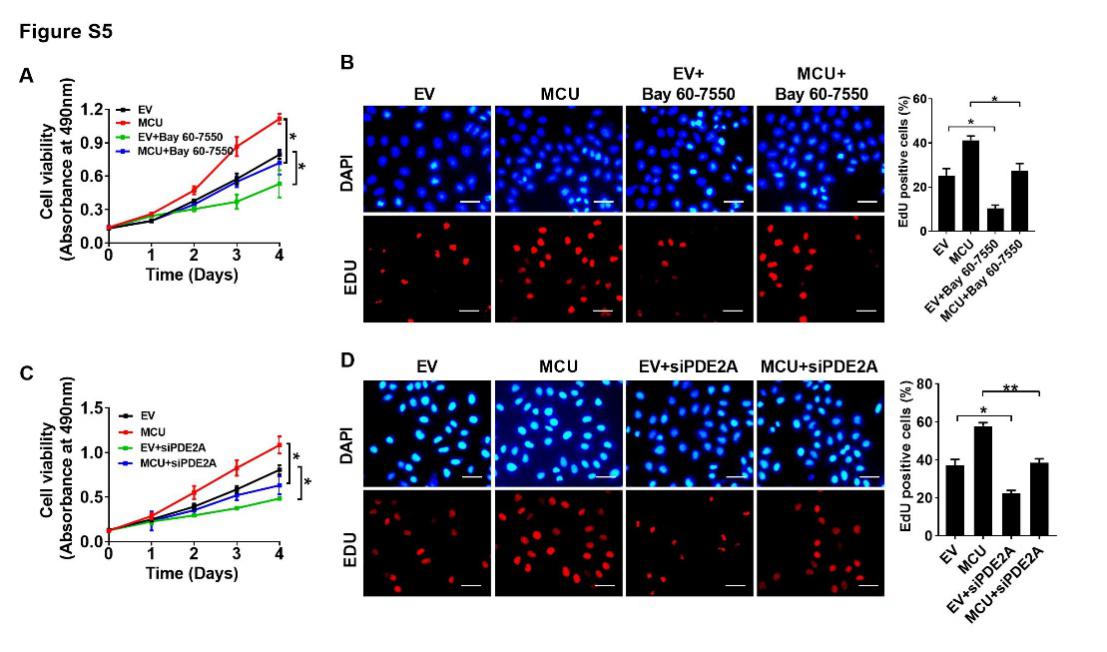


**Supplementary Figure 5**. **(A and B)** MTS assay for cell viability and **(C and D)** EdU incorporation assays for cell proliferation in FHC cells with treatments as indicated (scale bar, 20 μm). * *P<*0.05*;* ** *P<*0.01*.*


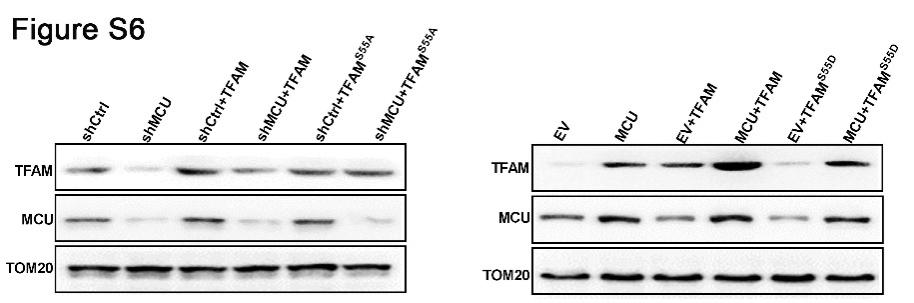


**Supplementary Figure 6. (A and B)** Western blot analysis for protein levels of mitochondrial MCU and TFAM in LS174T cells with treatments as indicated.

## Supplementary tables. Sequences of primers, siRNA and antibody information

**1.2.1 Primers used in gene cloning**

| **Gene** | **Primer pair** | **Primer sequence** |
| --- | --- | --- |
| *MCU* | Forward | GCGGATCCCGTTTCCAGTTGAGAGATGGCGGCC |
|  | Reverse | GCGAATTCGCCAGGATTCAGAGGCTTTTTGCAG |
| *PDE2A* | Forward | GCACCACATCCTCATCGCT |
|  | Reverse | AGACTCGCCCAGCGTCAC |
| *TFAM* | Forward | GCGGATCCATGGCGTTTCTCCGAAGCATGT |
|  | Reverse | GCGAATTCTTAACACTCCTCAGCACCATAT |

**1.2.2 siRNA**

| **siRNA** | **Primer pair** | **Primer sequence** |
| --- | --- | --- |
| siMCU | Sense | CUUCGACACUCAUGCCUUA |
|  | Anti-sense | UAAGGCAUGAGUGUCGAAG |
| siPKA | Sense | CAGGAAAGGGUAGAAUAAA |
|  | Anti-sense | UUUAUUCUACCCUUUCCUG |
| siPDE2A | Sense | GGAGCUGAUCUACAAAGAATT |
|  | Anti-sense | UUCUUUGUAGAUCAGCUCCTT |

**1.2.3 Primary antibodies used for western blot and immunohistochemistry.**

| **Antibody** | **Company (Cat.NO.)** | **Working dilutions** |
| --- | --- | --- |
| MCU | SIGMA (HPA05189) | WB: 1/200; |
| β–actin | TDY BIOTEC (TDY051C) | WB: 1/2000 |
| TFAM | Abcam (ab176558) | WB:1/1000 |
| Anti-Phosphoserine | Abcam (ab9332) | WB: 1/1000 |
| PDE2A | Abcam (ab224616) | WB: 1/1000 |
| Ki67 | MAIXIN-BIO (MAB-0542) | IHC:1/150 |
| PKAR2/PKR2 | Abcam (ab236855) | WB: 1/5000 |
| PRKACA | Proteintech (55388-1-AP) | WB: 1/500 |
| TOM20 | Proteintech (11802-1-AP) | WB: 1/5000 |
| GCAP1 | Abcam (100905) | WB: 1/1000 |
| sGC | Proteintech (17330-1-AP) | WB: 1/1000 |
| PKC | Abcam (ab181558) | WB: 1/1000 |
| p-PKA substrate | Cell signaling (#9624) | WB: 1/1000 |
